# Global trends and perspectives in mitophagy on neurodegenerative diseases: a scientometric analysis over 20 years

**DOI:** 10.3389/fmed.2025.1666909

**Published:** 2025-12-05

**Authors:** Haiyan Wu, Minheng Zhang, Lei Zhang, Hongwei Liu, Haixia Fan

**Affiliations:** 1 First Hospital of Shanxi Medical University, Taiyuan, Shanxi, China; 2 The First People’s Hospital of Jinzhong, Jinzhong, Shanxi, China; 3 Taiyuan City Central Hospital, Taiyuan, Shanxi, China

**Keywords:** mitophagy, neurodegenerative diseases, Alzheimer’s disease, mitochondrial dysfunction, oxidative stress, PINK1/Parkin

## Abstract

**Background:**

The investigation of mitophagy in neurodegenerative diseases has grown significantly, yet a comprehensive global insight remains limited. This study conducts a scientometric analysis to map the research landscape related to mitophagy in neurodegenerative diseases.

**Methods:**

We conducted a bibliometric analysis of 2,566 publications (2004 to 11 June 2025) from Web of Science Core Collection and Scopus. To mitigate bias in trend analyses, incomplete 2025 data were excluded from publication growth and curve fitting but retained for other analyses. Data were analyzed via Bibliometrix R package, VOSviewer, Scimago Graphica, and CiteSpace to map mitophagy research evolution.

**Results:**

The field showed exponential growth with peak productivity in 2021. The United States led publication output, with institutions from the USA, UK, and China forming the core of robust international collaborations, while maintaining the highest citation impact. Influential researchers included Tavernarakis, Nektarios and Reddy, P. Hemachandra, with prominent journals such as *International Journal of Molecular Sciences*, *Cells* and *Autophagy*, serving as key publication venues. Cluster analysis revealed thematic structures centered on “Parkinson’s disease,” “mitochondrial dysfunction,” “oxidative stress,” and “fission/fusion mechanisms”, with additional focus on “Parkin-mediated mitophagy” and “neurodegenerative diseases.” Research evolved from foundational studies through mechanistic exploration to translational applications. Emerging trends include “post-translational modifications (PTMs),” “chaperone-mediated autophagy,” “gut microbiota,” “mitochondrial quality control,” and therapeutic investigations of compounds like “curcumin” and “melatonin.”

**Conclusion:**

This first comprehensive scientometric analysis underscores the expanding interest in mitophagy as a crucial molecular mechanism in neurodegenerative diseases. Our findings establish a framework for developing novel therapeutic interventions such as mitochondrial quality control modulators and compounds like curcumin and melatonin targeting mitophagy dysfunction in neurodegenerative disorders.

## 1 Introduction

Mitophagy is a selective autophagic process aimed at removing damaged or dysfunctional mitochondria, thereby maintaining cellular homeostasis and reducing oxidative stress. It plays a critical role in cellular quality control and contributes to the prevention of aging and age-related disorders (1, 2). Recent studies have highlighted its importance in neurodegenerative diseases, suggesting that mitophagy can modulate disease progression through its effect on mitochondrial health (3–5).

Neurodegenerative diseases (ND), such as Alzheimer’s diseases (AD), Parkinson’s diseases (PD), and amyotrophic lateral sclerosis (ALS), are characterized by progressive neuronal loss and have been linked to a combination of genetic, environmental, and lifestyle factors (6, 7). These diseases often exhibit common pathogenic features, such as oxidative stress, mitochondrial dysfunction, and aberrant protein aggregation, which contribute to the progressive degeneration of the nervous system (8). Mitophagy is implicated in mitigating these pathologies by removing damaged mitochondria, thus preventing further cellular harm (9). The growing body of research highlights mitophagy as a promising therapeutic target for neurodegenerative disorders, aiming to restore cellular health by enhancing mitochondrial quality control mechanisms (6).

Bibliometric analysis is a powerful tool for evaluating the landscape of a scientific field. By analyzing citation patterns and research trends, bibliometric tools can provide insights into how a specific topic, such as mitophagy in ND, evolves over time. Techniques like co-citation analysis, co-word analysis, and science mapping are frequently used to identify key research areas, influential publications, and collaboration networks (10, 11). Systematic mapping is an emerging technique that evolves from systematic reviews, aiming to categorize research within a broad subject area. In this study, bibliometric methods were applied to assess the growth, influence, and collaboration patterns in the field of mitophagy research, shedding light on the development of scientific knowledge in this area (12, 13). To date, no scientometric analysis has focused specifically mitophagy on ND. This study aims to fill that gap by applying bibliometric methods to analyze and visualize relevant literature, summarize the field’s evolution, explore current research hotspots, and identify future research directions.

Our primary objective is to evaluate the evolution of mitophagy in ND over the past two decades, using co-citation network analysis to highlight pivotal studies and emerging trends. We also aim to map the research landscape by quantifying contributions from countries, institutions, authors, and journals. Ultimately, this analysis seeks to identify current gaps and opportunities, providing valuable insights for future research and policy development in ND.

## 2 Methods

### 2.1 Research design and scope

The data were retrieved from the Web of Science Core Collection (WoSCC) and Scopus from 2004 until the date of the search on 11 June 2025. Both databases were utilized to ensure comprehensive coverage and minimize the risk of missing relevant publications. WoSCC is a leading academic database recognized for its comprehensive coverage of scientific and authoritative publications, reliable citation indexing, and detailed citation information, including keywords and references, making it the most frequently used source in bibliometric studies (14, 15). Scopus complements WoSCC by indexing a broader range of journals, offering extensive coverage in the social sciences and life sciences, and providing robust author and affiliation level metrics that facilitate cross-disciplinary citation analyses and trend detection (14).

### 2.2 Search strategy and inclusion criteria

The study search used a combination of Medical Subject Headings (MeSH) terms and related entry terms sourced from PubMed. The keywords “mitophagy” and “neurodegenerative diseases” were employed with Boolean logic, with the complete search strategy, including all search terms and Boolean operators, as well as a flowchart illustrating the literature search, screening, and deduplication process, provided in the Supplementary file for transparency. This approach ensured that the search captured relevant articles discussing mitophagy in relation to ND. To refine the search results, built-in filters within the Web of Science and Scopus were applied to restrict the time span, document types, and languages. Only articles and reviews published in English were included in the final dataset. This filtering approach ensured the inclusion of high-quality and relevant publications, while maintaining focus on the most impactful research in the field.

### 2.3 Data processing

The collected data were first processed in R to identify and remove duplicate records, ensuring accuracy and uniqueness in the dataset. The processed data were analyzed using Bibliometrix and Excel for descriptive statistics, while VOSviewer, CiteSpace and and Scimago Graphica for the visualization of bibliometric networks. VOSviewer was primarily employed to construct and analyze co-authorship and co-citation networks. CiteSpace was also used for co-authorship and co-citation network analysis, as well as for generating theme clusters, cluster timelines, and detecting keyword bursts. Scimago Graphica was utilized specifically to generate international collaboration maps. These tools facilitated the identification of major research trends, contributing countries, influential institutions, key researchers, seminal publications, and significant keywords and themes in the field of mitophagy and ND.

### 2.4 Data analysis

In the analyses conducted, different tools were employed to extract and process the data. The Bibliometrix R package (4.4.1) (16) were utilized to gather information on countries/regions, journals, and authors. For bibliometric analyses, including the examination of co-citation and co-occurrence networks, software tools VosViewer (1.6.19) (17), CiteSpace (6.4.R1) (18) and Scimago Graphica(1.0.52) (19) were deployed.

## 3 Results

### 3.1 Annual publications and citations growth

**FIGURE 1:**
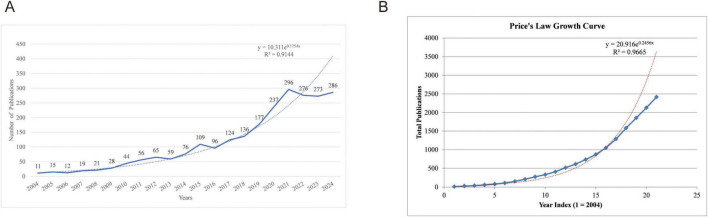
Publication trend. **(A)** Annual publication growth trends; **(B)** Price’s Law analysis of accumulative publication counts.

The annual publication is one of the important indicators for evaluating the development of scientific research, and to a certain extent, it reflects the growth of knowledge in the relevant field. Over the period from 2004 to 2024, which excluded incomplete 2025 data to avoid bias in recent trends, a total of 2,416 publications related to mitophagy in neurodegenerative diseases were analyzed. As illustrated in Figure 1A, the number of published articles has exhibited a generally consistent upward trajectory, reaching a peak of 296 publications in 2021. Although subsequent years exhibited fluctuations—276 in 2022, 273 in 2023, and 286 in 2024—these may be partially attributable to database indexing delays, particularly for the more recent years (2023–2024). Nonetheless, these observations do not obscure the overall pattern of sustained growth in publication output, indicating the field’s robust academic expansion. Besides, a generalized additive model was utilized to evaluate the correlation between publication counts and the years they were published. The analysis demonstrated that the model was accurate in aligning with the yearly publication trend, as indicated by an *R*^2^ of 0.9144. To investigate the temporal growth pattern of literature, the Price’s Law exponential growth model was applied to the cumulative number of publications. As shown in Figure 1B, the total number of publications exhibited a clear upward trend over time, suggesting a potential exponential growth pattern. After excluding publication data from 2025 to eliminate potential outliers or incomplete records, an exponential regression model was constructed using sequential year indices (where *x* = 1 corresponds to 2004, *x* = 2 to 2005, and so on up to *x* = 21 for 2024) rather than absolute years to avoid numerical scaling issues. The resulting regression equation was: *y* = 20.916*e*^0^.^2456*x*^, with a coefficient of determination *R*^2^ = 0.9665, indicating an excellent fit and underscoring accelerating interest in mitophagy’s role in ND.

### 3.2 Analysis of the most productive countries

**FIGURE 2:**
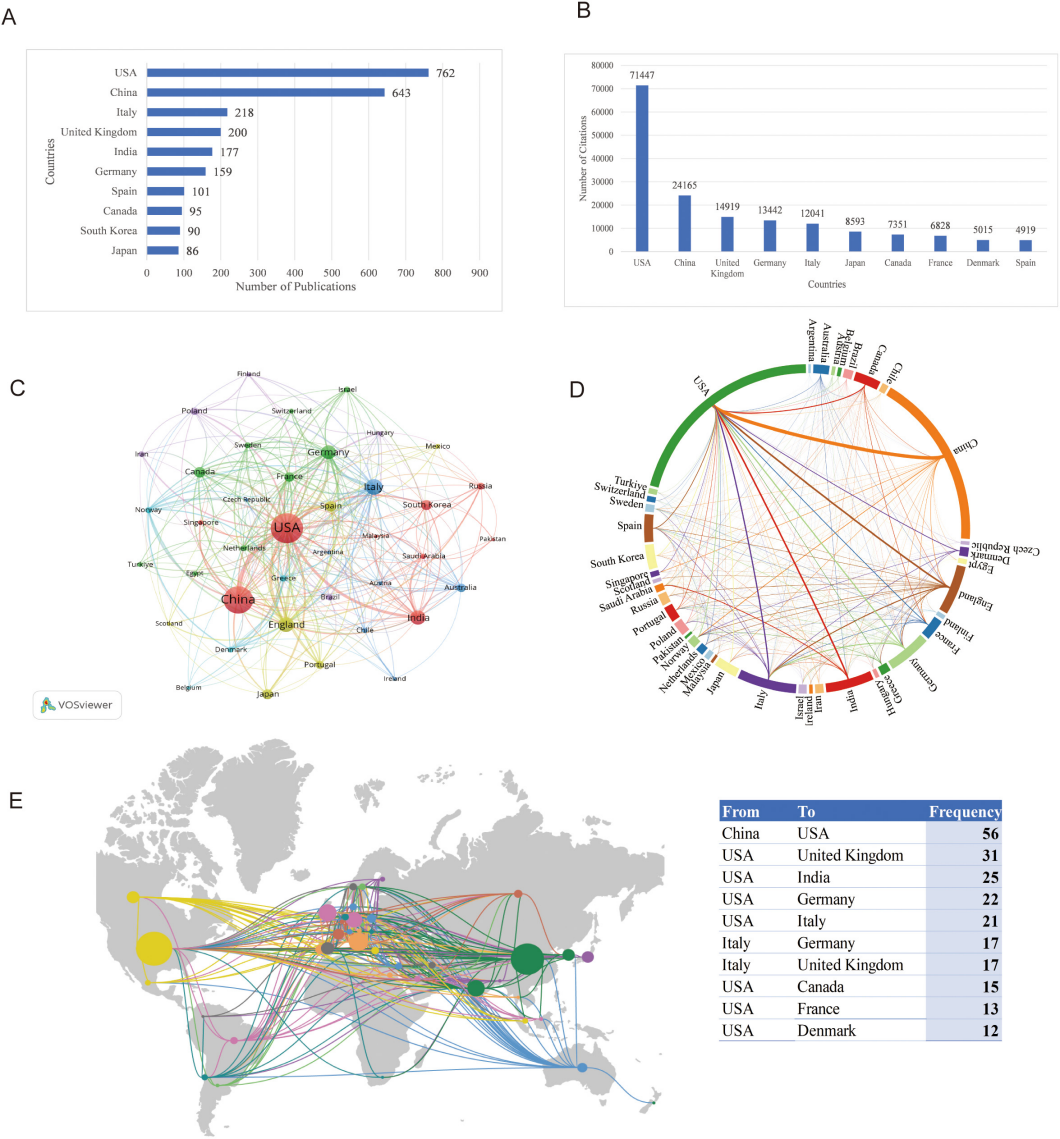
Analysis of countries. **(A)** Top 10 countries by publication output; **(B)** Top 10 countries by citation count; **(C)** Network visualization of international research collaborations by VOSviewer; **(D)** Chord diagram of country collaborative relationships; **(E)** Geographical mapping of major collaboration flows with accompanying table showing the most frequent country partnerships.

A total of 105 countries contributed to research on mitophagy in neurodegenerative diseases. As shown in Figure 2A, the top 10 countries or regions by publication count are presented. The USA was the most productive country with 762 publications. Researchers from China and Italy contributed 643 and 218 articles, respectively, ranking second and third. In terms of citations illustrated in Figure 2B, the USA also led with 71,447 citations, followed by China with 24,165 citations, while the United Kingdom ranked third with 12,041 citations. Figures 2C–E illustrated the research collaborations among various countries and regions, highlighting that the majority of partnerships occur among the top research-producing countries, particularly between the USA, China, European nations such as the UK and Italy and other developed countries. Notably, the collaboration between USA and China was the most frequent, with a recorded frequency of 56 instances. This was followed by the collaborative efforts between the United States and the United Kingdom (31), and between the United States and India (25), which were respectively ranked second and third. These collaboration patterns aligned with countries’ publication outputs, suggesting that nations with strong research capabilities often took a leading role. Such leadership not only enhanced collaborative opportunities but also facilitated bidirectional knowledge transfer, particularly between established research powers and emerging scientific communities.

### 3.3 Analysis of the most productive institutions

**FIGURE 3:**
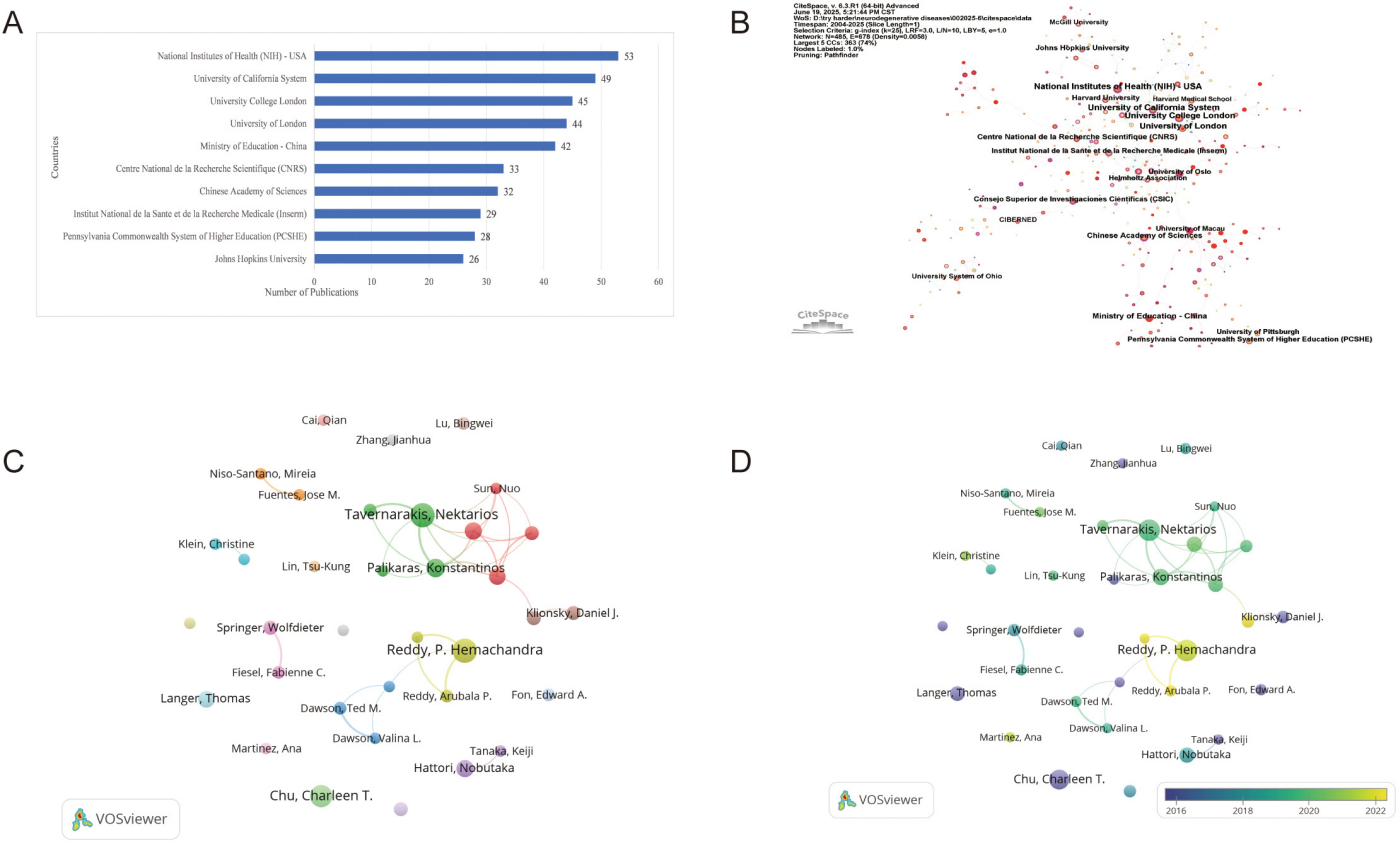
Analysis of research institutions and authors. **(A)** Top 10 research institutions by publication output; **(B)** Network visualization of institutional collaborations from CiteSpace; **(C)** Co-authorship network visualized in VOSviewer; **(D)** Co-authorship overlay network;

A total of 3,186 institutions worldwide were involved in mitophagy research in neurodegenerative diseases. Figure 3A showed the publication output of leading institutions, with the National Institutes of Health (NIH) leading with 53 publications, followed by the University of California System (49) and University College London (45). American and European institutions dominated the rankings, with notable contributions from Chinese institutions. Supplementary Table 1 provided multi-dimensional analysis beyond publication counts. Network centrality analysis revealed the Chinese Academy of Sciences as the most influential research hub (centrality = 0.19), followed by NIH (0.17), University of California System (0.14), and University College London (0.11).

Temporally, most institutions began significant contributions around 2007–2008, though the Ministry of Education of China entered later in 2014, reflecting expanding international interest. Burst analysis identified periods of intense research activity (citation), with the University of London demonstrating the highest research momentum (burst = 7.7756), followed by the Ministry of Education of China (7.3554) and University College London (6.9315). Collaboration analysis (Figure 3B) revealed a network of 485 institutions with 678 collaborative links and a density of 0.0058, indicating sparse collaboration with significant potential for expansion. Collaboration serves as a catalyst for innovation, facilitating interdisciplinary integration and knowledge exchange among institutions (20). The network structure confirmed that high-centrality institutions like NIH, University of California System, and University College London not only produced substantial research output but also served as key catalysts connecting the global research community. These findings collectively demonstrated that institutional leadership in this field encompassed publication volume, network influence, and collaborative connectivity.

### 3.4 Analysis of the most productive authors

Authors serve as the driving force behind scientific publication production, making the analysis of their contributions a critical indicator of field advancement (21). Among the 12,369 authors who contributed to this field, Tavernarakis Nektarios ranked first with 18 publications and 2,032 citations (h-index = 13). He investigates mitophagy dysfunction’s role in driving age-related neurodegenerative diseases like Alzheimer’s (22), and molecular links between mitophagy, cellular homeostasis, and longevity, emphasizing how maintaining mitophagic flux counters neural pathologies (23, 24). He was closely followed by Reddy, P. Hemachandra, who also had 18 publications but fewer citations (1,248, h-index = 12); Reddy, P. Hemachandra studies mitochondrial abnormalities in neurodegenerative diseases, focusing on oxidative damage from free radicals (25), fission/fusion dynamics linked to synaptic harm (e.g., Aβ-Drp1 in Alzheimer’s), and shared metabolic pathways with obesity and diabetes (26). Chu, Charleen T. ranked third with 16 publications and demonstrated higher citation influence (1,653 citations, h-index = 15), as her work specializes selective autophagy and mitophagy in neurodegenerative diseases like Parkinson’s, focusing on how impaired mitochondrial clearance contributes to neuronal damage (27). Her research explores kinase signaling and mitochondrial quality control, emphasizing neuroprotection strategies (28) (Supplementary Table 3).

The author collaboration networks (Figure 3C) revealed comprehensive insights into the structural organization of the research community. Most remarkably, one prominent collaborative cluster emerged, containing five of the top ten authors. Tavernarakis, Nektarios demonstrated the highest collaborative activity (Total Link Strength: 25), followed by Palikaras, Konstantinos (20) and Fang, Evandro F. (18), establishing these three as primary collaborative drivers. Bohr, Vilhelm A. showed moderate engagement (13), while Klionsky, Daniel J. maintained minimal connections (1). This cluster represented a powerhouse of the research field, combining high productivity with exceptional citation impact. The remaining top author, Reddy, P. Hemachandra, led a separate cluster with substantial collaborative activity (16), whereas Chu, Charleen T., Langer, Thomas, and Holzbaur, Erika L. F. operated independently (Total Link Strength: 0). Hattori, Nobutaka demonstrated minimal collaborative engagement (3). Additionally, the overlay visualization in Figure 3D revealed distinct temporal patterns when combined with the start year data from Supplementary Table 3. The clusters demonstrated varying maturity levels: early pioneers like Hattori, Nobutaka (2004), Langer, Thomas (2006), and Klionsky, Daniel J. (2009) established foundational frameworks, appearing in blue-colored zones. Researchers from the establishment phase, such as Tavernarakis, Nektarios (2013), and Palikaras, Konstantinos (2015), occupied transitional zones, reflecting their integration during the field’s expansion. Most remarkably, recent researchers Bohr, Vilhelm A. and Fang, Evandro F. (both 2017) appeared in the light-yellow zones, indicating their emergence during the latest phase of mitophagy research.

### 3.5 Analysis of highly-impacted journals

Journal analysis effectively assists researchers in identifying the most relevant publications for their studies and interests, thereby enhancing the quality of their research evaluations (29). Supplementary Table 4 listed the top 10 journals among 698 publications from various dimensions in the field. The leading contributors were *International Journal of Molecular Sciences*, *Cells*, and *Autophagy*, which contributed 114 (4.44%), 69 (2.69%), and 55 (2.14%) papers on mitophagy in neurodegenerative diseases, respectively.

**FIGURE 4:**
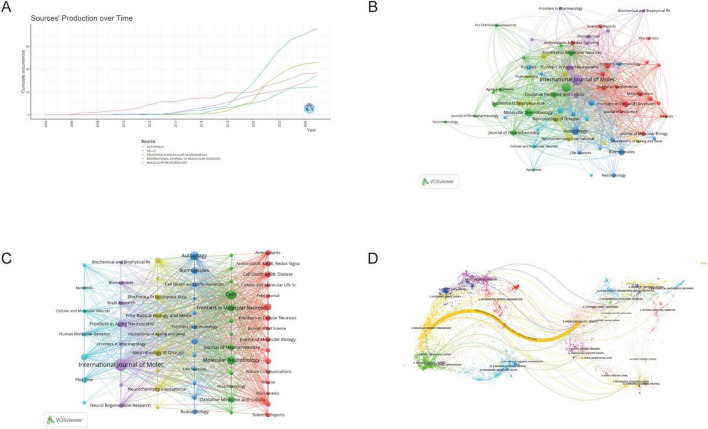
Analysis of journals. **(A)** Annual publication trends of the top five journals; **(B)** Journal Connections and Collaboration Networks; **(C)** Journal co-citation visualization analysis; **(D)** Dual-map overlay analysis (colored spots on the left side of the map represent citing journals from different disciplines, while the colored spots on the right side correspond to cited journals).

Figure 4A showed the temporal development of the top five journals in the field, demonstrating consistent growth in recent years and reflecting an overall upward trend. Among the top 10 journals, 50% were classified as Q1 quartile and 50% as Q2 quartile. Three journals achieved impact factors greater than 5, with *Autophagy* standing out with an exceptional impact factor of 14.6, reflecting its significant influence in the field. Figure 4B presented the interconnections among these journals.

The listed journals covered a broad spectrum of biological and neuroscience disciplines, providing diverse platforms for publishing mitophagy research in neurodegenerative diseases. From the specialized focus of *Autophagy* to the comprehensive molecular perspective of *Journal of Biological Chemistry*, these publications collectively offered a comprehensive landscape for exploring the molecular, cellular, and neurobiological aspects of mitophagy. *Frontiers in Neuroscience* and *Neurobiology of Disease* were particularly relevant, as they directly addressed the neurological implications of mitophagy.

In terms of co-citation analysis, Figure 4C highlighted *Journal of Biological Chemistry* (7,919), *Proceedings of the National Academy of Sciences of the United States of America* (7,167), and *Nature* (6,127) as the most frequently co-cited journals, each having been cited more than 6,000 times. Figure 4D presented the dual-map overlay visualization depicting citation relationships between citing and cited journals. The yellow path indicated that citing publications primarily originated from journals in “molecular biology and genetics,” while cited papers predominantly derived from journals in “molecular biology and immunology.” This analysis revealed the knowledge development patterns and research directions in the field.

### 3.6 Literature analysis

#### 3.6.1 Literature citation analysis

**FIGURE 5:**
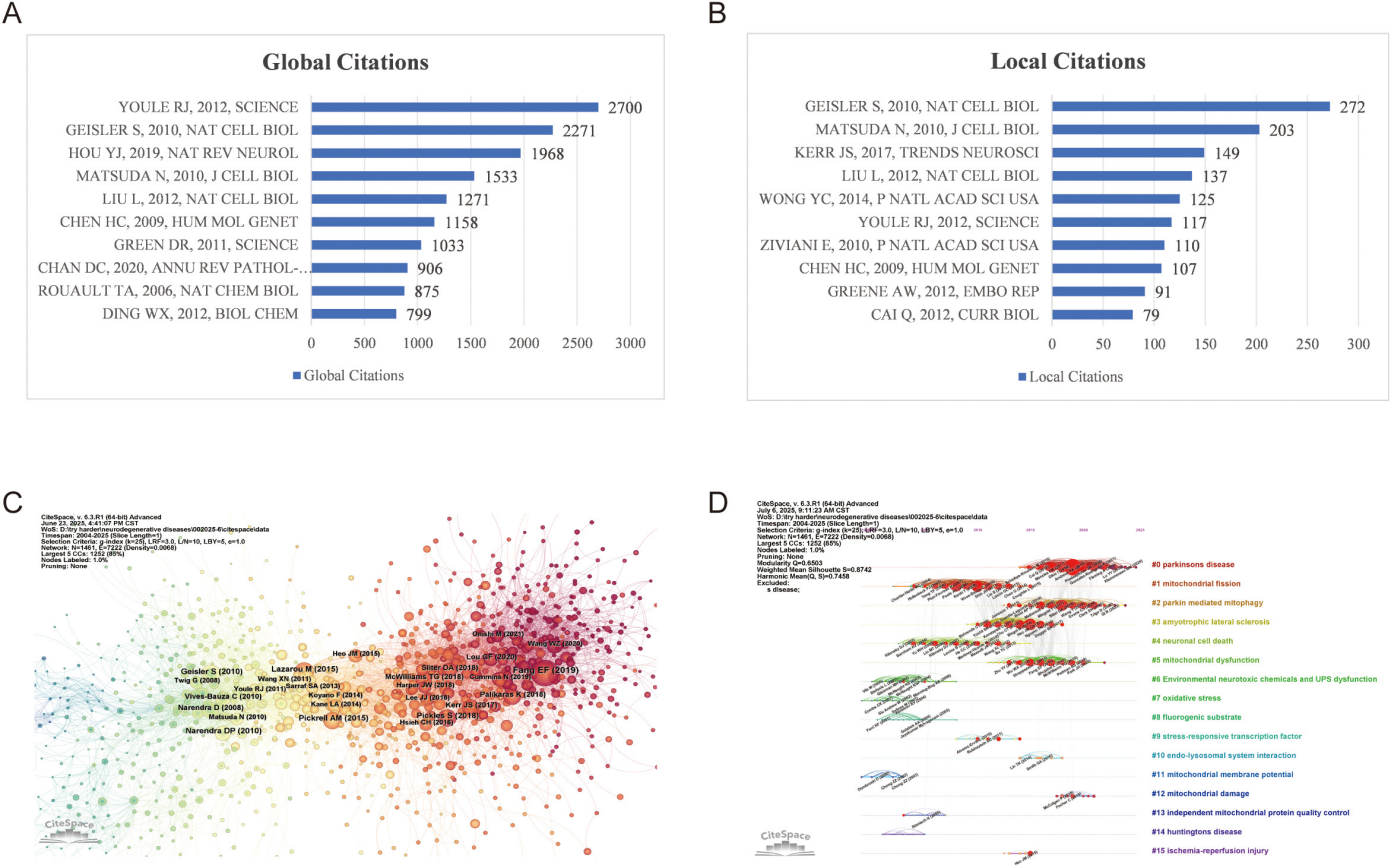
Analysis of literature. **(A)** Top 10 most global cited literatures; **(B)** Top 10 most local cited literatures; **(C)** Co-citation reference network generated by CiteSpace; **(D)** Co-citation reference clustering timeline produced by CiteSpace;

To identify the most influential papers within these 2,566 publications, both local and global citation counts were analyzed, as shown in Figures 5A, B. The paper by Youle et al. (30) received the highest global citation count with 2,700 times. This influential review examined mitochondrial dynamics (fission, fusion, and stress responses) in cellular homeostasis. Fusion enables functional complementation while fission facilitates removal of damaged mitochondria via mitophagy. The work emphasized the PINK1–Parkin pathway, where PINK1 accumulates on damaged mitochondria to recruit Parkin for autophagic clearance, linking these processes to neurodegenerative diseases like Parkinson’s. The paper by Geisler et al. (31)ranked first in local citations (2,271) and second in global citations (272). This influential study examined the roles of PINK1 (PTEN-induced kinase 1) and Parkin, two key proteins in mitophagy, and explored how their activities were regulated through interactions with VDAC1 (voltage-dependent anion channel 1) and p62/SQSTM1, a receptor protein that binds ubiquitinated proteins.

Besides, co-citation refers to a measure of the relationship between two documents, determined by how frequently they are cited together in other works (32). It serves as an important tool for identifying and tracking research fronts as well as the intellectual foundations of a field (18). Figure 5C illustrated the most co-cited references from the 159,877 references across 2,566 publications included in this study, while Supplementary Table 5 presented detailed information about top 10 frequently co-cited references. Fang et al. (22) had the highest co-citation frequency at 211, followed by Lazarou et al. (33) with 111, and Pickles et al. (34) with 105. Fang et al. (22) demonstrated that stimulating mitophagy could reverse cognitive deficits and inhibit amyloid-β and tau pathology in both cellular and animal models of AD, highlighting defective mitophagy as a key driver of AD progression through cross-species evidence (human samples, C. elegans, and mouse models) and therapeutic strategies like urolithin A and NAD + precursors. Lazarou et al. (33) highlighted how PINK1 interacted with autophagy receptors, facilitating their recruitment to damaged mitochondria for degradation, revealing a Parkin-independent mechanism with implications for neurodegenerative diseases such as ALS and glaucoma linked to receptor mutations. Pickles et al. (34) discussed the mechanisms by which cells eliminated damaged or dysfunctional mitochondria to prevent cellular stress and maintain energy homeostasis, focusing on the roles of PINK1 and Parkin in PD pathogenesis, including mitochondrial quality control defects in multi-species models and clinical associations.

#### 3.6.2 Literature cluster analysis

The co-citation relationships and documents were analyzed from the cluster and timeline evaluation perspective, and 16 largest clusters were detected from the 2,566 publications and their 159,877 references, resulting in a density of 0.0068. The network demonstrated a modularity of 0.6503, indicating a well-defined structure, and a weighted mean silhouette of 0.8742, which signified a high-quality clustering configuration. Figure 5D displayed 16 key clusters in timeline view (#0 Parkinson’s disease, #1 mitochondrial fission, #2 parkin mediated mitophagy, #3 amyotrophic lateral sclerosis, #4 neuronal cell death, #5 mitochondrial dysfunction, #6 environmental neurotoxic chemicals-induced UPS dysfunction, #7 oxidative stress, #8 fluorogenic substrate, #9 stress-responsive transcription factor, #10 endo-lysosomal system interaction, #11 mitochondrial membrane potential, #12 mitochondrial damage, #13 independent mitochondrial protein quality control, #14 Huntington’s disease, #15 ischemia-reperfusion injury). The timeline view illustrated the development of each theme from its early stages to the present, as indicated along the top. The start, peak, and decline of each theme were clearly visible, with current focuses primarily on Cluster #0 parkinsons disease, and Cluster #2 parkin mediated mitophagy. Clusters were arranged vertically, from largest to smallest, and their citation relationships were shown by colored lines within and between clusters. The large red nodes were noteworthy, as they represent references that were either highly influential, had experienced citation bursts, or both. Below each timeline, the most frequently cited papers were displayed.

### 3.7 Keywords analysis

#### 3.7.1 Keywords frequency analysis

Keywords are important indicators of publication content and can be used to explain and understand the centrality of research themes, or hotspots, within a specific field (35).

**FIGURE 6:**
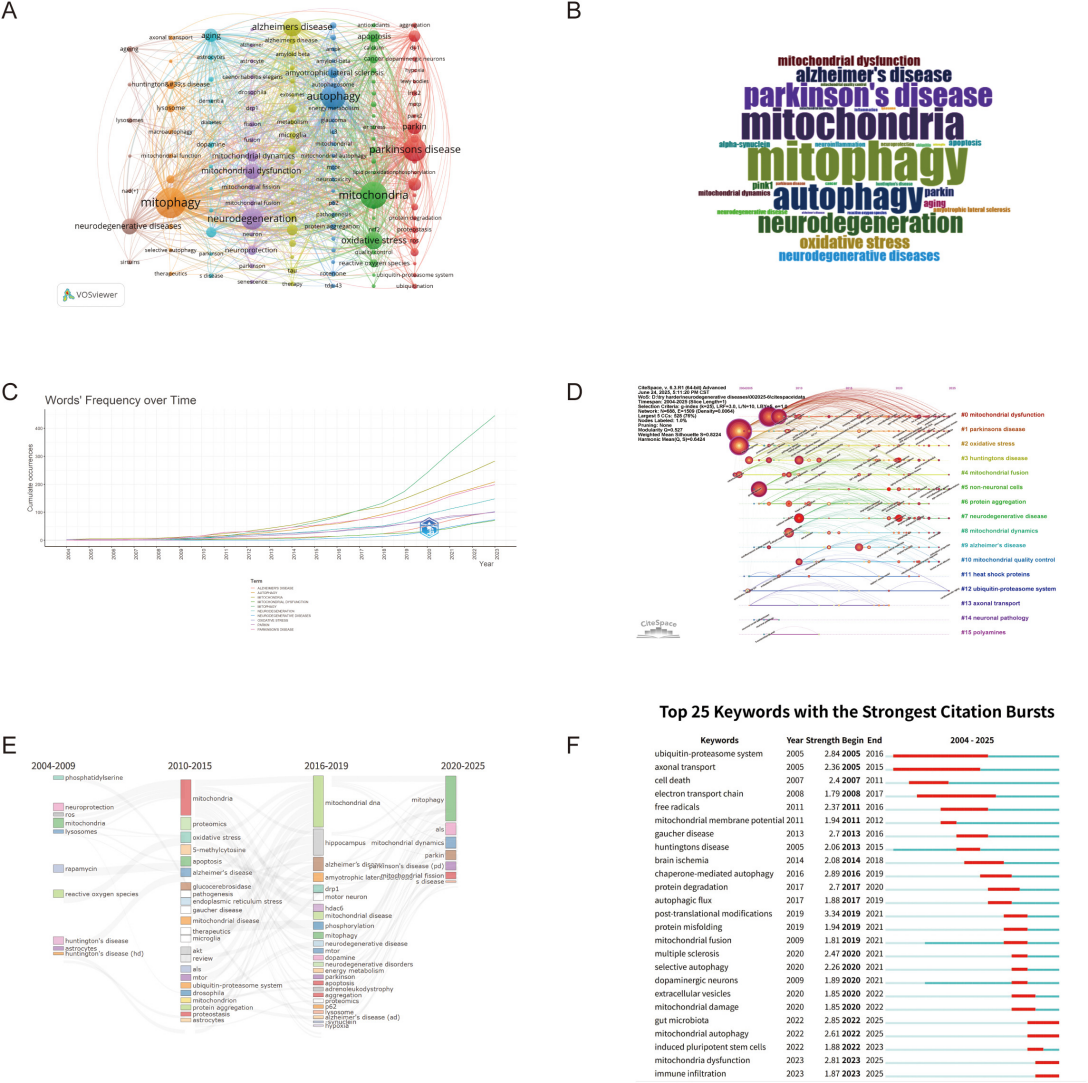
Analysis of keywords. **(A)** Keywords co-occurrence network visualization from VosViewer; **(B)** Word cloud of keywords from Biblimetrix package; **(C)** Development of the top 10 most frequent keywords over time; **(D)** Keywords clustering timeline visualization from CiteSpace; **(E)** Theme evaluation from Biblimetrix package; **(F)** Top 25 keywords with the strongest citation bursts based on CiteSpace.

A total of 4,144 author keywords were identified, of which 138 had a frequency of more than 10 occurrences, as shown in Figure 6A created using VosViewer. The frequency analysis of these keywords was also presented as a word cloud in the Figure 6B. Among the most frequent keywords, “mitophagy” ranked first, followed by “mitochondria” and “autophagy,” which were the second and third most frequent keywords, respectively. The next most frequent terms were “Parkinson’s disease,” “neurodegeneration,” “Alzheimer’s disease,” “oxidative stress,” “neurodegenerative diseases,” “mitochondrial dysfunction,” and “Parkin.” Additionally, the development of the top 10 keywords over the last 21 years was also illustrated in Figure 6C.

#### 3.7.2 Keywords cluster analysis

Keyword cluster analysis provides valuable insights into the field. As shown in Figure 6D, the timeline visualization of the top 16 keyword clusters was generated using CiteSpace. The LLR algorithm was applied to label each cluster presented in Supplementary Table 6. The modularity Q was 0.527, reflecting a strong community structure within the network. The weighted mean silhouette value of 0.8224, further demonstrated excellent cluster cohesion and separation. The largest clusters identified were mitochondrial dysfunction (#0), which appeared as the most prominent research focus with the largest node size, followed by Parkinson’s disease (#1), oxidative stress (#2), Huntington’s disease (#3), and mitochondrial fusion (#4). These primary clusters represent the core research themes in the field, with mitochondrial dysfunction serving as the fundamental concept connecting various neurodegenerative conditions. Additional significant clusters included non-neuronal cells (#5), protein aggregation (#6), neurodegenerative disease (#7), and mitochondrial dynamics (#8). The timeline visualization reveals the temporal evolution of research focus, with earlier studies (2004–2010) establishing foundational concepts in mitochondrial dysfunction and oxidative stress, while more recent research (2018–2023) has expanded into specialized areas such as mitochondrial quality control (#10), the ubiquitin-proteasome system (#12), and neuronal pathology (#14).

To understand how the keywords evolved over time, the thematic evolution function from the Bibliometrix package in R was utilized, as illustrated in Figure 6E. The 21-year period was divided into three stages: 2004–2009, 2010–2015, and 2010–2025. Each block in the figure represented a theme, with different colors distinguishing various themes. The term adjacent to each block indicated the major keyword characterizing the respective theme. Links between blocks reflected the evolution of themes over time, with the strength of the links indicating the degree of connection between the themes.

Figure 6F illustrated the top 25 keywords with the strongest citation bursts, offering critical insights into the evolution of research themes over the past two decades. Among these, “post-translational modifications” (2019–2021, burst strength: 3.34) demonstrated the highest burst strength, and it was followed by “chaperone-mediated autophagy” (2016–2019, burst strength: 2.89) and “gut microbiota” (2022–2025, burst strength: 2.86) as the top three keywords. Long-standing keywords, which maintained relevance over extended periods, include “ubiquitin-proteasome system” (2005–2016), “axonal transport” (2005–2015), and “electron transport chain” (2008–2017). In contrast, the most recent research hotspots include keywords such as “mitochondrial autophagy,” “gut microbiota,” “mitochondria dysfunction” and “immune infiltration” which highlight emerging areas of interest.

## 4 Discussion

Mitophagy stands as a core driver in the pathogenesis of diverse neurodegenerative diseases, with its dysfunction emerging as a unifying pathogenic thread across these disorders. The persistent accumulation of oxidative stress—arising from mitochondrial dysfunction—further aggravates neuronal damage, making it a central focus in the study of PD, AD, and HD. Drawing on our bibliometric insights and the broader literature, we synthesize below the core molecular frameworks, disease-specific mechanisms, and emerging research frontiers that shape the current understanding of mitophagy in neurodegenerative diseases.

### 4.1 Shared molecular pathways underlying mitophagy: the PINK1–Parkin axis, mitochondrial dynamics, and proteostasis regulation

The central role of mitophagy in neurodegenerative diseases is primarily driven by the PINK1-Parkin pathway, which has emerged as the key framework in the field, particularly in PD. Mutations in PINK1 or Parkin impair this pathway, leading to mitochondrial dysfunction, the accumulation of ROS, and neuronal death (36). This pathway operates through a defined cascade: PINK1 accumulates on damaged mitochondria, phosphorylates ubiquitin at Ser65, and recruits Parkin to initiate the ubiquitination of outer mitochondrial membrane proteins for degradation (37).

Mitochondrial dynamics, particularly the balance between fission and fusion, plays a vital role in mitochondrial quality control and cellular function. DRP1-mediated fission segregates damaged mitochondria for mitophagy, while MFN1/2-mediated fusion allows partially dysfunctional mitochondria to recover (38). In HD, excessive mitochondrial fission, driven by mutant huntingtin, results in fragmented mitochondria and impaired energy production, while in AD, amyloid-beta and tau proteins disrupt mitochondrial dynamics, worsening mitochondrial function (38). These disruptions are especially damaging in neurons with long axons, such as dopaminergic neurons in PD and motor neurons in ALS, where fragmented mitochondria fail to efficiently deliver ATP to distal segments, leading to energy depletion and dysfunction (39). Therapeutic attempts to modulate mitochondrial dynamics have encountered challenges, as inhibitors like Mdivi-1, which target DRP1, may impair physiological fission necessary for mitophagy, highlighting the need for precision in restoring mitochondrial dynamics (39). Disruptions in calcium homeostasis, further exacerbated by mitochondrial dynamics dysfunction, complicate potential therapeutic strategies.

Proteostasis, the balance of protein folding, degradation, and clearance, is another interconnected pathway involved in mitochondrial dysfunction. The UPS tags damaged or misfolded proteins for degradation, while CMA transports specific substrates to lysosomes (40). In PD, Parkin functions as a dual E3 ubiquitin ligase, tagging both mitochondrial proteins for mitophagy and cytosolic α-synuclein for proteasomal degradation. Mutations impair the UPS, leading to the accumulation of α-synuclein and toxic aggregates (40, 41). This creates a bidirectional pathology: α-synuclein aggregates overload the UPS and inhibit CMA by blocking LAMP2A multimerization at the lysosomal membrane (42). Loss of DJ-1 function further impairs CMA, increasing α-synuclein levels and neurotoxicity, particularly in aging individuals where LAMP2A expression declines (42). In HD, mutant huntingtin disrupts CMA, impairing protein clearance and accelerating neurodegeneration. This proteostasis failure, which may involve the simultaneous impairment of the UPS, CMA, and mitophagy, suggests that HD may require multi-pathway therapeutic interventions, distinguishing it from PD, where the PINK1-Parkin pathway plays a more central role.

### 4.2 Mitophagy: a double-edged sword in neurodegeneration

Mitophagy maintains neuronal homeostasis by clearing damaged mitochondria, reducing ROS, and preventing apoptosis. Yet, excessive or impaired mitophagy disrupts energy balance and synaptic function. In Parkinson’s and Alzheimer’s diseases, mutations in PINK1, Parkin, and related genes upset this balance, leading to neurodegeneration. Thus, mitophagy acts as a double-edged sword: protective when properly regulated, yet detrimental when excessive, requiring a balance that demands precise modulation to preserve mitochondrial integrity and neuronal homeostasis.

In AD, disrupted mitochondria-associated membranes (MAMs) impair mitochondrial function and promote Aβ accumulation, linking mitochondrial dysfunction to AD pathogenesis (43). Defective mitophagy exacerbates neuronal injury through enhanced Aβ and Tau aggregation, oxidative stress, and neuroinflammation (44, 45). Decreased NLRX1 expression leads to abnormal mitochondrial morphology and increased Aβ levels in APP/PS1 models, highlighting NLRX1 as a potential therapeutic target (46). Pharmacological enhancement of mitophagy, such as Remimazolam-mediated activation of the PINK1/Parkin pathway, restores mitochondrial function and reduces Aβ_1–42_-induced cytotoxicity and Tau hyperphosphorylation (47). Moreover, mitochondrial aging and dysregulated dynamics (fission/fusion imbalance) contribute to bioenergetic failure and Aβ42 accumulation, while stress-induced BNIP3L upregulation may restore mitophagy efficiency (48–50). Together, these findings illustrate how mitophagy in AD can be both neuroprotective and pathogenic, depending on its degree and timing of activation.

In PD, mitophagy dysfunction is central to dopaminergic neuron degeneration. Mutations such as VPS35 p.D620N impair PINK1/Parkin-mediated mitophagy and enhance LRRK2 kinase activity, accelerating disease progression (51). Accumulation of α-synuclein at MAMs disrupts mitochondrial dynamics, further inhibiting mitophagy and promoting oxidative stress (43). Pharmacological interventions that restore mitophagy—such as Erbai decoction, FGF21, ORA471, and Asiatic acid—have been shown to alleviate mitochondrial dysfunction and improve motor performance in PD models (52–55). While moderate activation of mitophagy protects neuronal integrity, overactivation may deplete mitochondria, reinforcing the concept that mitophagy in PD must be finely balanced (56).

In HD, the accumulation of mutant huntingtin (mHTT) disrupts mitochondrial quality control, leading to bioenergetic deficits and neuronal death. Impaired mitophagy is a hallmark of HD pathology, associated with defective autophagy mechanisms and the toxic interaction between mHTT and voltage-dependent anion channel 1 (VDAC1), which hinders mitochondrial clearance (57, 58). Inhibition of ROCK2 aggravates oxidative stress and neurodegeneration, underscoring the importance of mitophagy regulation (59). Conversely, pharmacological modulation using SCH79797 can prevent 3-NP-induced mitophagy via the PINK1/ubiquitin pathway, highlighting its protective role (60). These findings indicate that controlled enhancement of mitophagy may alleviate mitochondrial dysfunction and neurodegeneration in HD, whereas its dysregulation can accelerate pathology.

In ALS, mitophagy impairment contributes to motor neuron degeneration through mitochondrial dysfunction and oxidative stress. The bioflavonoid isoginkgetin enhances PINK1/Parkin-dependent mitophagy, improving mitochondrial health and motor neuron survival (61). Conversely, inhibition of ROCK2 and loss of OPTN reduce autophagic flux, leading to ROS accumulation and neuronal injury (59, 62). Structural abnormalities and altered expression of mitophagy-related genes have been observed in SOD1-G93A mice, further linking mitophagy dysregulation to ALS progression (63). Moreover, increased m6A RNA methylation downregulates TFEB expression, impairing mitophagy, whereas TFEB overexpression rescues mitochondrial function (64). These studies reinforce the dual nature of mitophagy in ALS: essential for maintaining mitochondrial integrity yet detrimental when imbalanced.

Overall, across neurodegenerative diseases such as AD, PD, HD, and ALS, mitophagy emerges as a finely tuned but fragile process. Its precise temporal and spatial regulation determine whether it serves as a protector of neuronal homeostasis or a promoter of degeneration, underscoring the therapeutic need to calibrate rather than simply enhance or inhibit mitophagy.

### 4.3 Emerging hotspots: post-translational modifications and gut microbiota

Recent advances in PTMs have become a prominent focus in the study of mitophagy regulation. Ubiquitination, in particular, has been identified as a key PTM driving the recruitment of autophagy receptors like NDP52 and OPTINEURIN, which facilitate the degradation of damaged mitochondria (65). This has opened new avenues for therapeutic interventions, such as small molecules like kinetin riboside, which can activate the PINK1-Parkin pathway independently of mitochondrial depolarization (66).

Additionally, USP30, a deubiquitinase that antagonizes Parkin, represents a promising target, with inhibitors such as MTX115 undergoing clinical trials (67). Unraveling the regulatory roles of these PTMs in mitophagy and mitochondrial homeostasis opens new avenues for targeted therapy, establishing this area as a rapidly advancing research frontier in the field.

The role of the gut microbiota in modulating mitophagy has emerged as a novel research hotspot. Gut dysbiosis—imbalance in gut microbial populations—impairs intestinal barrier function, allowing bacterial lipopolysaccharides to enter the bloodstream, activating peripheral immune cells. These pro-inflammatory cytokines cross the blood-brain barrier and activate microglia, which then exhibit defective mitophagy, leading to ROS accumulation and the activation of inflammasomes (68). This creates a feedback loop, exacerbating neuronal injury.

On the other hand, beneficial microbial metabolites such as short-chain fatty acids have been shown to positively modulate mitophagy. For instance, butyrate enhances PINK1/Parkin-mediated mitophagy and alleviates symptoms in PD models (69), while urolithin A, derived from gut microbiota, induces mitophagy and improves cognitive function in AD models (70).

### 4.4 Future directions

Future studies on mitophagy are expected to move toward multi-omics integration, spatiotemporal imaging, and translational research. Multi-omics approaches—combining genomics, proteomics, and metabolomics—will enable comprehensive mapping of mitophagy regulatory networks and their alterations across disease progression. Advances in super-resolution and live-cell imaging, such as two-photon microscopy and fluorescence lifetime imaging, will facilitate the visualization of mitochondrial dynamics in real time, offering unprecedented insights into the cellular mechanisms underlying neurodegeneration.

Furthermore, integrating artificial intelligence with systems biology modeling holds great promise for decoding the intricate regulatory networks of mitophagy and pinpointing actionable therapeutic targets. The development of mitophagy-modulating agents—such as small molecules enhancing PINK1/Parkin signaling or mitophagy-inducing peptides—represents a promising avenue for disease intervention, bridging basic research and clinical translation.

### 4.5 Limitations

This study provides the first comprehensive bibliometric evaluation of mitophagy research in neurodegenerative diseases, offering quantitative insights into its evolution, thematic structure, and emerging trends. We have established an objective and reproducible analytical framework to identify key contributors and research clusters. However, several limitations should be acknowledged. First, reliance on the Web of Science Core Collection, Scopus, and English-language publications may introduce selection bias, potentially omitting significant contributions from non-English or non-indexed journals. Second, inconsistencies in author and institutional naming conventions, as well as publication delays, could influence bibliometric accuracy. Third, while keyword and cluster analyses effectively reveal research patterns, they cannot fully capture the nuanced conceptual depth or experimental diversity within the field. Therefore, integrating bibliometric analyses with systematic reviews and meta-analyses will offer a more holistic perspective on the evolving research landscape of mitophagy in neurodegenerative diseases.

## 5 Conclusion

To the best of our knowledge, this study represents the first comprehensive examination of mitophagy in ND research trends using a bibliometric approach. Over the past two decades, the field has experienced substantial growth, with 2,566 publications recorded from 2004 to 2025 and a peak in 2021 (followed by a slight dip in 2022–2023), underscoring the expanding importance of mitophagy in understanding and treating neurodegenerative disorders. Our analysis demonstrates that mitophagy is a critical molecular mechanism in ND research and holds significant potential as a therapeutic target for addressing mitochondrial dysfunction and oxidative stress.

The United States emerged as the most productive country (762 publications) and, together with China and Italy, forms the core of a robust international collaboration network. Key institutions—particularly the U.S. National Institutes of Health, the University of London, and University College London—drive this research, while leading authors such as Tavernarakis Nektarios and Reddy P. Hemachandra (18 publications each) and Bohr Vilhelm A. (extraordinarily high citations) have shaped its trajectory. Journals like International Journal of Molecular Sciences, Cells, and Autophagy serve as primary outlets, reflecting both high publication volume and impact. Keyword analysis highlights “mitophagy,” “mitochondria,” and “Parkinson’s disease” as central themes, mirroring the field’s growing scope.

Moreover, this study introduces a detailed evaluation of trends, influential authors, institutions, journals, and collaboration networks within the field, providing a novel perspective for bibliometric analysis in ND research. By analyzing co-citations, keyword trends, and cluster evolution, we present a roadmap for identifying key areas of interest and potential research directions. In conclusion, our findings broaden the understanding of the relationship between mitophagy, neurodegenerative mechanisms, and therapeutic interventions, offering a theoretical framework for developing novel clinical-trial drugs targeting mitophagy. This innovative approach aims to inspire future researchers to adopt bibliometric methods for uncovering emerging topics and advancing scientific understanding, ultimately contributing to the development of innovative therapeutic approaches for ND treatment.

## Data Availability

The original contributions presented in this study are included in this article/Supplementary material, further inquiries can be directed to the corresponding author.
