# Supplementary material for: Global trends and perspectives in mitophagy on neurodegenerative diseases: a scientometric analysis over 20 years
Source: Front Med (Lausanne). 2025 Dec 5;12:1666909. doi: 10.3389/fmed.2025.1666909 (PMC12714877; doi:10.3389/fmed.2025.1666909)
Supplement: Supplementary file 1 [file Data_Sheet_1.docx]

Global Trends and Perspectives in Mitophagy on Neurodegenerative Diseases:

A Scientometric Analysis over 20 Years

Running title: Mitophagy in ND

**Supplementary material:**

**Table S1. Search strategy.**

**Table S2. Top 10 institutions of mitophagy in neurodegenerative diseases.**

**Table S3. Top 10 authors of mitophagy in neurodegenerative diseases.**

**Table S4. Top 10 journals in mitophagy in neurodegenerative diseases.**

**Table S5. Top 10 co-citation references in neurodegenerative diseases.**

**Table S6. Clusters with more than 100 references and current research focus clusters.**

**Figure S1. Literature selection flowchart.**

**Table S1. Search strategy.**

| **Database** | **Search Strategy** |
| --- | --- |
| Scopus | ((TITLE-ABS-KEY (“Mitophagy”) OR TITLE-ABS-KEY (“Mitochondrial Degradation”))) AND ((TITLE-ABS-KEY (“Neurodegenerative Diseases”) OR TITLE-ABS-KEY (“Neurodegenerative Disease”) OR TITLE-ABS-KEY (“Degenerative Neurologic Disorders”) OR TITLE-ABS-KEY (“Degenerative Neurologic Disorder”) OR TITLE-ABS-KEY (“Neurologic Disorder, Degenerative”) OR TITLE-ABS-KEY (“Neurologic Disorders, Degenerative”) OR TITLE-ABS-KEY (“Nervous System Degenerative Diseases”) OR TITLE-ABS-KEY (“Neurodegenerative Disorders”) OR TITLE-ABS-KEY (“Neurodegenerative Disorder”) OR TITLE-ABS-KEY (“Degenerative Diseases, Nervous System”) OR TITLE-ABS-KEY (“Degenerative Diseases, Neurologic”) OR TITLE-ABS-KEY (“Neurologic Degenerative Disease”) OR TITLE-ABS-KEY (“Neurologic Degenerative Conditions”) OR TITLE-ABS-KEY (“Degenerative Condition, Neurologic”) OR TITLE-ABS-KEY (“Degenerative Conditions, Neurologic”) OR TITLE-ABS-KEY (“Neurologic Degenerative Condition”) OR TITLE-ABS-KEY (“Neurologic Degenerative Diseases”) OR TITLE-ABS-KEY (“Degenerative Neurologic Diseases”) OR TITLE-ABS-KEY (“Degenerative Neurologic Disease”) OR TITLE-ABS-KEY (“Neurologic Disease, Degenerative”) OR TITLE-ABS-KEY (“Neurologic Diseases, Degenerative”) OR TITLE-ABS-KEY (“Degenerative Diseases, Central Nervous System”) OR TITLE-ABS-KEY (“Degenerative Diseases, Spinal Cord”))) |
| Web of Science Core Collection | (TS=(Neurodegenerative Diseases) OR TS=(Neurodegenerative Disease) OR TS=(Degenerative Neurologic Disorders) OR TS=(Degenerative Neurologic Disorder) OR TS=(Neurologic Disorder, Degenerative) OR TS=(Neurologic Disorders, Degenerative) OR TS=(Nervous System Degenerative Diseases) OR TS=(Neurodegenerative Disorders) OR TS=(Neurodegenerative Disorder) OR TS=(Degenerative Diseases, Nervous System) OR TS=(Degenerative Diseases, Neurologic) OR TS=(Neurologic Degenerative Disease) OR TS=(Neurologic Degenerative Conditions) OR TS=(Degenerative Condition, Neurologic) OR TS=(Degenerative Conditions, Neurologic) OR TS=(Neurologic Degenerative Condition) OR TS=(Neurologic Degenerative Diseases) OR TS=(Degenerative Neurologic Diseases) OR TS=(Degenerative Neurologic Disease) OR TS=(Neurologic Disease, Degenerative) OR TS=(Neurologic Diseases, Degenerative) OR TS=(Degenerative Diseases, Central Nervous System) OR TS=(Degenerative Diseases, Spinal Cord)) AND (TS=(Mitophagy) OR TS=(Mitochondrial Degradation)) |

**Table S2. Top 10 institutions of mitophagy in neurodegenerative diseases.**

| Institutions | Count | Centrality | Start Year | Burst | Country |
| --- | --- | --- | --- | --- | --- |
| National Institutes of Health (NIH) - USA | 53 | 0.17 | 2005 | 3.4675 | USA |
| University of California System | 49 | 0.14 | 2007 | 0.05 | USA |
| University College London | 45 | 0.11 | 2008 | 6.9315 | UK |
| University of London | 44 | 0.05 | 2008 | 7.7756 | UK |
| Ministry of Education - China | 42 | 0.09 | 2014 | 7.3554 | China |
| Centre National de la Recherche Scientifique (CNRS) | 33 | 0.09 | 2007 | 0.05 | France |
| Chinese Academy of Sciences | 32 | 0.19 | 2011 | 0.05 | China |
| Institut National de la Sante et de la Recherche Medicale (Inserm) | 29 | 0.11 | 2005 | 0.05 | France |
| Pennsylvania Commonwealth System of Higher Education (PCSHE) | 28 | 0 | 2007 | 3.7994 | USA |
| Johns Hopkins University | 26 | 0.03 | 2004 | 0.05 | USA |

**Table S3. Top 10 authors of mitophagy in neurodegenerative diseases.**

| Author | Documents | Citations | Total Link Strength | h_index | g_index | m_index | Start Year |
| --- | --- | --- | --- | --- | --- | --- | --- |
| Tavernarakis, Nektarios | 18 | 2032 | 25 | 13 | 18 | 1 | 2013 |
| Reddy, P. Hemachandra | 18 | 1248 | 16 | 12 | 19 | 0.857 | 2012 |
| Chu, Charleen T. | 16 | 1653 | 0 | 15 | 16 | 0.789 | 2007 |
| Palikaras, Konstantinos | 13 | 1523 | 20 | 11 | 14 | 1 | 2015 |
| Bohr, Vilhelm A. | 12 | 3335 | 13 | 10 | 13 | 1.111 | 2017 |
| Hattori, Nobutaka | 12 | 2161 | 3 | 10 | 13 | 0.455 | 2004 |
| Fang, Evandro F. | 11 | 2145 | 18 | 16 | 19 | 1.778 | 2017 |
| Langer, Thomas | 11 | 1603 | 0 | 11 | 11 | 0.55 | 2006 |
| Holzbaur, Erika L. F. | 9 | 1686 | 0 | 10 | 11 | 0.833 | 2014 |
| Klionsky, Daniel J. | 9 | 2012 | 1 | 8 | 9 | 0.471 | 2009 |

**Table S4. Top 10 journals in mitophagy in neurodegenerative diseases.**

| Sources | Articles | Citations | Average Citation | IF | JCR |
| --- | --- | --- | --- | --- | --- |
| International Journal of Molecular Sciences | 114 | 1995 | 17.50 | 4.9 | Q1 |
| Cells | 69 | 2988 | 43.30 | 5.1 | Q2 |
| Autophagy | 55 | 1283 | 23.33 | 14.6 | Q1 |
| Molecular Neurobiology | 52 | 887 | 17.06 | 4.6 | Q2 |
| Frontiers in Molecular Neuroscience | 37 | 462 | 12.49 | 3.5 | Q2 |
| Biomolecules | 35 | 689 | 19.69 | 4.8 | Q1 |
| Neurobiology of Disease | 34 | 1057 | 31.09 | 5.1 | Q1 |
| Frontiers in Aging Neuroscience | 33 | 939 | 28.45 | 4.1 | Q2 |
| Frontiers in Cell and Developmental Biology | 33 | 617 | 18.70 | 4.6 | Q1 |
| Journal of Biological Chemistry | 33 | 1570 | 47.58 | 4.0 | Q2 |

**Table S5. Top 10 co-citation references in neurodegenerative diseases.**

| Rank | | Co-citation Counts | Year | Title | | Authors | Journal |
| --- | --- | --- | --- | --- | --- | --- | --- |
| 1 | | 211 | 2019 | Mitophagy inhibits amyloid-β and tau pathology and reverses cognitive deficits in models of Alzheimer's disease[1] | | Fang E F, Hou Y, Palikaras K, et al. | Nature neuroscience |
| 2 | 111 | | 2015 | The ubiquitin kinase PINK1 recruits autophagy receptors to induce mitophagy[2] | | Lazarou M, Sliter D A, Kane L A, et al. | Nature |
| 3 | 105 | | 2015 | The roles of PINK1, parkin, and mitochondrial fidelity in Parkinson's disease[3] | | Pickrell A M, Youle R J. | Neuron |
| 4 | 104 | | 2018 | Mitophagy and quality control mechanisms in mitochondrial maintenance[4] | Pickles S, Vigié P, Youle R J. | | Current Biology |
| 5 | 95 | | 2010 | PINK1 is selectively stabilized on impaired mitochondria to activate Parkin[5] | Narendra D P, Jin S M, Tanaka A, et al. | | PLoS biology |
| 6 | 94 | | 2010 | PINK1/Parkin-mediated mitophagy is dependent on VDAC1 and p62/SQSTM1[6] | Geisler S, Holmström K M, Skujat | | Nature cell biology |
| 7 | 87 | | 2018 | Mechanisms of mitophagy in cellular homeostasis, physiology and pathology[7] | Kerr J S, Adriaanse B A, Greig N H, et al. | | Trends in neurosciences |
| 8 | 86 | | 2008 | Parkin is recruited selectively to impaired mitochondria and promotes their autophagy[8] | Narendra D, Tanaka A, Suen D F, et al. | | The Journal of cell biology |
| 9 | 85 | | 2017 | Mitophagy and Alzheimer's disease: cellular and molecular mechanisms[9] | Kerr J S, Adriaanse B A, Greig N H, et al. | | Trends in neurosciences |
| 10 | 75 | | 2018 | Basal mitophagy occurs independently of PINK1 in mouse tissues of high metabolic demand[10] | McWilliams T G, Prescott A R, Montava-Garriga L, et al. | | Cell metabolism |

**Table S6. Clusters with more than 100 references and current research focus clusters.**

| Cluster ID | Size | Silhouette | Mean Year | Most Cited Reference | Major Citing Article |
| --- | --- | --- | --- | --- | --- |
| 0 | 284 | 0.859 | 2019 | Fang, E. F. *et al.* Mitophagy inhibits amyloid-beta and tau pathology and reverses cognitive deficits in models of Alzheimer's disease (2019)[1] | Montava-Garriga, L. & Ganley, I. G. Outstanding Questions in Mitophagy: What We Do and Do Not Know (2020)[11] |
| 1 | 236 | 0.840 | 2009 | Narendra, D. P. *et al.* PINK1 is selectively stabilized on impaired mitochondria to activate Parkin (2010)[5] | Lionaki, E., Markaki, M., Palikaras, K. & Tavernarakis, N. Mitochondria, autophagy and age-associated neurodegenerative diseases: New insights into a complex interplay (2015)[12] |
| 2 | 190 | 0.777 | 2018 | Pickles, S., Vigie, P. & Youle, R. J. Mitophagy and Quality Control Mechanisms in Mitochondrial Maintenance (2018)[4] | Montava-Garriga, L. & Ganley, I. G. Outstanding Questions in Mitophagy: What We Do and Do Not Know (2020)[11] |
| 3 | 135 | 0.890 | 2013 | Lazarou, M. *et al.* The ubiquitin kinase PINK1 recruits autophagy receptors to induce mitophagy (2015)[2] | Bingol, B. & Sheng, M. Mechanisms of mitophagy: PINK1, Parkin, USP30 and beyond (2016)[13] |
| 4 | 133 | 0.956 | 2007 | Hara, T. *et al.* Suppression of basal autophagy in neural cells causes neurodegenerative disease in mice (2006)[14] | Wong, A. S., Cheung, Z. H. & Ip, N. Y. Molecular machinery of macroautophagy and its deregulation in diseases (2011)[15] |
| 5 | 103 | 0.857 | 2016 | Wei, Y., Chiang, W. C., Sumpter, R., Jr., Mishra, P. & Levine, B. Prohibitin 2 Is an Inner Mitochondrial Membrane Mitophagy Receptor (2017)[16] | Liu, L. *et al.* Mitophagy and Its Contribution to Metabolic and Aging-Associated Disorders (2020)[17] |
| 12 | 11 | 0.988 | 2019 | Wang, P. *et al.* TDP-43 induces mitochondrial damage and activates the mitochondrial unfolded protein response (2019)[18] | Xu, S. *et al.* Dual roles of UPR(er) and UPR(mt) in neurodegenerative diseases(2023)[19] |


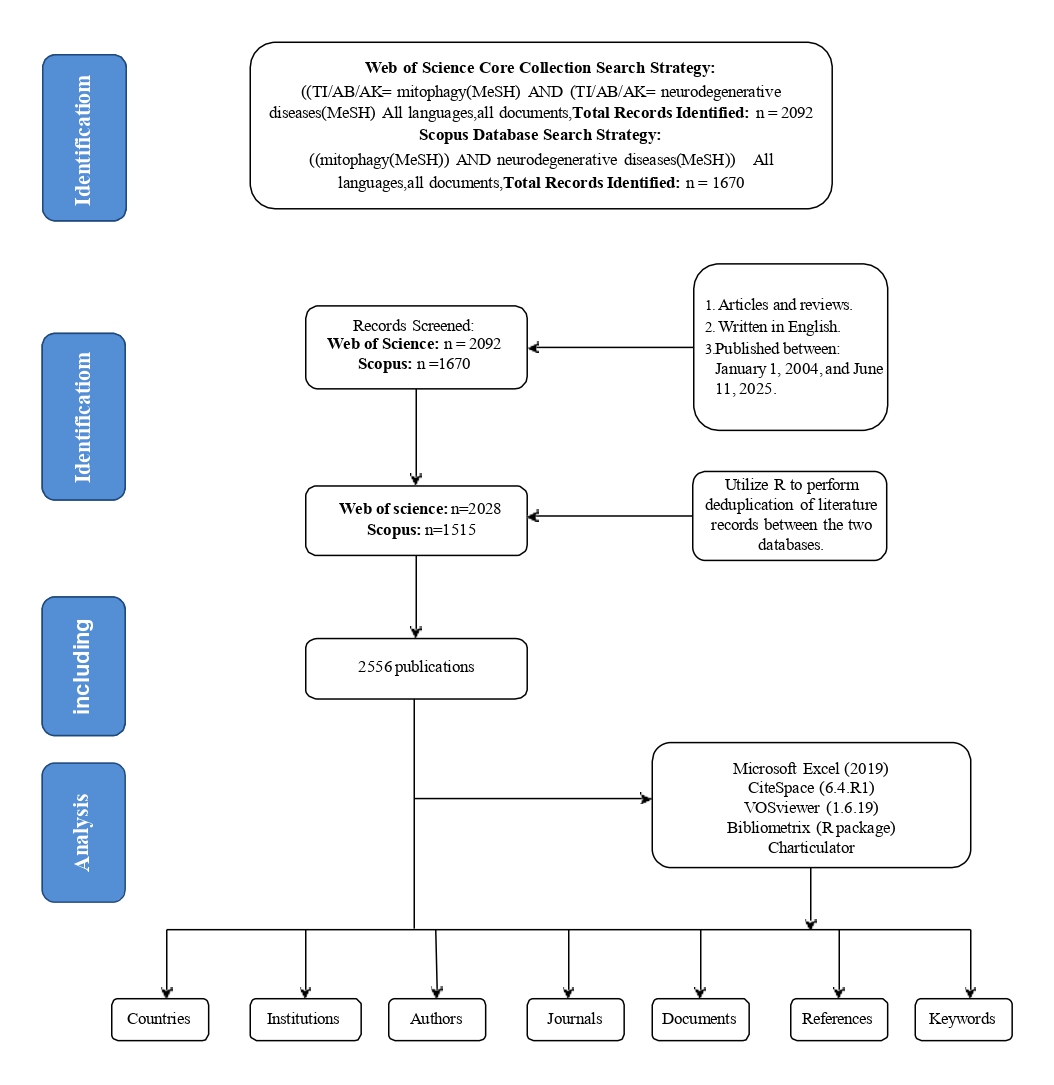
**Figure S1. Literature selection flowchart.**

[1]. Fang EF, Hou Y, Palikaras K*, et al.* Mitophagy inhibits amyloid-beta and tau pathology and reverses cognitive deficits in models of Alzheimer's disease. *Nat Neurosci*. 2019 **22:** 401-412.

[2]. Lazarou M, Sliter DA, Kane LA*, et al.* The ubiquitin kinase PINK1 recruits autophagy receptors to induce mitophagy. *Nature*. 2015 **524:** 309-314.

[3]. Pickrell AM, Youle RJ. The roles of PINK1, parkin, and mitochondrial fidelity in Parkinson's disease. *Neuron*. 2015 **85:** 257-273.

[4]. Pickles S, Vigie P, Youle RJ. Mitophagy and Quality Control Mechanisms in Mitochondrial Maintenance. *Curr Biol*. 2018 **28:** R170-R185.

[5]. Narendra DP, Jin SM, Tanaka A*, et al.* PINK1 is selectively stabilized on impaired mitochondria to activate Parkin. *PLoS Biol*. 2010 **8:** e1000298.

[6]. Geisler S, Holmstrom KM, Skujat D*, et al.* PINK1/Parkin-mediated mitophagy is dependent on VDAC1 and p62/SQSTM1. *Nat Cell Biol*. 2010 **12:** 119-131.

[7]. Palikaras K, Lionaki E, Tavernarakis N. Mechanisms of mitophagy in cellular homeostasis, physiology and pathology. *Nat Cell Biol*. 2018 **20:** 1013-1022.

[8]. Narendra D, Tanaka A, Suen DF, Youle RJ. Parkin is recruited selectively to impaired mitochondria and promotes their autophagy. *J Cell Biol*. 2008 **183:** 795-803.

[9]. Kerr JS, Adriaanse BA, Greig NH*, et al.* Mitophagy and Alzheimer's Disease: Cellular and Molecular Mechanisms. *Trends Neurosci*. 2017 **40:** 151-166.

[10]. McWilliams TG, Prescott AR, Montava-Garriga L*, et al.* Basal Mitophagy Occurs Independently of PINK1 in Mouse Tissues of High Metabolic Demand. *Cell Metab*. 2018 **27:** 439-449 e435.

[11]. Montava-Garriga L, Ganley IG. Outstanding Questions in Mitophagy: What We Do and Do Not Know. *J Mol Biol*. 2020 **432:** 206-230.

[12]. Lionaki E, Markaki M, Palikaras K, Tavernarakis N. Mitochondria, autophagy and age-associated neurodegenerative diseases: New insights into a complex interplay. *Biochim Biophys Acta*. 2015 **1847:** 1412-1423.

[13]. Bingol B, Sheng M. Mechanisms of mitophagy: PINK1, Parkin, USP30 and beyond. *Free Radic Biol Med*. 2016 **100:** 210-222.

[14]. Hara T, Nakamura K, Matsui M*, et al.* Suppression of basal autophagy in neural cells causes neurodegenerative disease in mice. *Nature*. 2006 **441:** 885-889.

[15]. Wong AS, Cheung ZH, Ip NY. Molecular machinery of macroautophagy and its deregulation in diseases. *Biochim Biophys Acta*. 2011 **1812:** 1490-1497.

[16]. Wei Y, Chiang WC, Sumpter R, Jr., Mishra P, Levine B. Prohibitin 2 Is an Inner Mitochondrial Membrane Mitophagy Receptor. *Cell*. 2017 **168:** 224-238 e210.

[17]. Liu L, Liao X, Wu H, Li Y, Zhu Y, Chen Q. Mitophagy and Its Contribution to Metabolic and Aging-Associated Disorders. *Antioxid Redox Signal*. 2020 **32:** 906-927.

[18]. Wang P, Deng J, Dong J*, et al.* TDP-43 induces mitochondrial damage and activates the mitochondrial unfolded protein response. *PLoS Genet*. 2019 **15:** e1007947.

[19]. Xu S, Liu H, Wang C*, et al.* Dual roles of UPR(er) and UPR(mt) in neurodegenerative diseases. *J Mol Med (Berl)*. 2023 **101:** 1499-1512.
